# Rapid prediction of wall shear stress in stenosed coronary arteries based on deep learning

**DOI:** 10.3389/fbioe.2024.1360330

**Published:** 2024-08-12

**Authors:** Salwa Husam Alamir, Vincenzo Tufaro, Matilde Trilli, Pieter Kitslaar, Anthony Mathur, Andreas Baumbach, Joseph Jacob, Christos V. Bourantas, Ryo Torii

**Affiliations:** ^1^ Department of Mechanical Engineering, University College London, London, United Kingdom; ^2^ Barts Heart Centre, Barts Health NHS Trust, London, United Kingdom; ^3^ Centre for Cardiovascular Medicine and Devices, William Harvey Research Institute, Queen Mary University of London, London, United Kingdom; ^4^ Medis Medical Imaging Systems BV, Leiden, Netherlands; ^5^ NIHR Barts Biomedical Research Centre, Queen Mary University of London, London, United Kingdom; ^6^ Satsuma Lab, Centre for Medical Image Computing, University College London, London, United Kingdom; ^7^ UCL Respiratory, University College London, London, United Kingdom

**Keywords:** deep learning, coronary artery, stenosis, computational fluid dynamics, synthetic data

## Abstract

There is increasing evidence that coronary artery wall shear stress (WSS) measurement provides useful prognostic information that allows prediction of adverse cardiovascular events. Computational Fluid Dynamics (CFD) has been extensively used in research to measure vessel physiology and examine the role of the local haemodynamic forces on the evolution of atherosclerosis. Nonetheless, CFD modelling remains computationally expensive and time-consuming, making its direct use in clinical practice inconvenient. A number of studies have investigated the use of deep learning (DL) approaches for fast WSS prediction. However, in these reports, patient data were limited and most of them used synthetic data generation methods for developing the training set. In this paper, we implement 2 approaches for synthetic data generation and combine their output with real patient data in order to train a DL model with a U-net architecture for prediction of WSS in the coronary arteries. The model achieved 6.03% Normalised Mean Absolute Error (NMAE) with inference taking only 0.35 s; making this solution time-efficient and clinically relevant.

## 1 Introduction

Coronary Artery Disease (CAD) is the leading cause of death globally and is associated with approximately 9 million deaths worldwide (Khan et al., 2020). Local hemodynamic forces distribution and in particular wall shear stress (WSS) seems to play a pivotal role in the initiation of the atherosclerotic process and its evolution. Measurement of WSS can predict vulnerable plaques and adverse cardiovascular events (Stone et al., 2018). Blood vessels with large diameters and low flow are exposed to low WSS, whereas vessels with a small lumen and high flow are exposed to high WSS (Cecchi et al., 2011). As *in vivo* experimental measurement of WSS is impractical, computational “measurement” using computational fluid dynamics (CFD) has been broadly used to assess coronary physiology. Whilst the efficiency of CFD modelling has improved over the last decades, these models are still computationally expensive and time-consuming, limiting their direct use in clinical practice (Ferdian et al., 2022). To address this challenge, several studies have investigated the use of deep learning (DL) approaches for fast WSS prediction.

DL has commonly been used in the medical imaging domain for fast data analysis. An example in the field of flow modelling is in image-based estimation of boundary conditions for blood flow problems, assisted by DL (Arzani et al., 2022). DL has also been used for biomedical problems in various stages of numerical modelling; some of which include automatic generation and quality assessment of meshes (Zhang et al., 2020).

Moreover, DL has also been used to predict numerical simulation results. For instance, Gharleghi et al. (2022) applied a DL solution to predict time-varying WSS in the left main coronary bifurcation while Jordanski et al. (2018) used DL methods and in particular multivariate linear regression, multilayer perceptron and Gaussian conditional random fields in order to predict WSS distribution at the carotid bifurcation. Furthermore, Suk et al. (2022) utilised mesh convolutional neural networks in order to predict WSS in a 3D coronary artery with and without bifurcation using synthetically generated coronary arteries with stenosis.

These models are mostly inspired by fully convolutional networks (FCN) in combination with autoencoders (encoder-decoder models) that were initially developed for image segmentation (Ronneberger et al., 2015). The architectures that have become well known are U-net and V-net, with U-net being the most adopted (Shen et al., 2017). Similar to autoencoders, a U-net architecture consists of one part that contracts to capture global context, followed by a second part to expand and therefore enable localisation. In the study of Gharleghi the application of U-net for DL- WSS prediction et al. enabled WSS estimation of coronary left main stem bifurcation with a normalised mean absolute error (Gharleghi et al., 2020) of 10.38% (with 0.56% std.), based on 3,429 training data sets including patient-specific (127) and synthetic data (3,302).

However, DL-based prediction of WSS in stenosed coronary arteries using patient-specific geometries has not widely been studied, despite the fact that these are potentially highly clinically-relevant analyses. An earlier study used multi-layer perceptrons, multivariate linear regression, and convolutional neural networks to generate WSS values from 2,000 patient-based but idealized coronary artery geometry (Su et al., 2020). In this paper, for the first time, we apply a U-net-based DL prediction method to extract WSS in real patients’ stenosed coronary arteries. It is known that the training of a U-net, similar to other deep learning models, relies on a large dataset. As such, the implementation of data augmentation methods in advance can be used in order to learn effectively from very few annotated data samples. As patient data for training is limited in this study, we used simple methods to generate synthetic data for training and evaluate method’s efficacy.

## 2 Materials and methods

### 2.1 Patient data

#### 2.1.1 Data source and patient characteristics

Stenosed coronary artery geometry and centreline data derived from X-ray angiograms and CFD simulation results were obtained from our previous study (Tufaro et al., 2021). Fifty vessels from the study cohort were randomly extracted for the present analysis and their geometries are displayed in Supplementary Figure S7. The original study included patients that had a coronary angiogram for clinical purposes between January 2012 and June 2017 from three cardiac centres in the United Kingdom: Barts Heart Centre (London), Essex Cardiothoracic Centre (Basildon) and Royal Free Hospital (London). The dataset consisted of patients who underwent a coronary angiography and had at least one intermediate atherosclerotic lesion with a fractional flow reserve (FFR) of 0.81–0.85. Exclusion criteria included an ambiguous culprit lesion in the context of an acute coronary syndrome presentation, lesions at the edge of a stent ( < 5 mm), lesions at the ostium of the right coronary artery or the left main stem. Cases with angiographic projections that were less than 25° apart were also excluded, as adequate three-dimensional coronary reconstruction from the angiographic data is not possible. The local ethics committee advised that a formal ethical approval was not required for the conduction of the present research. The baseline demographics of the study patient are summarised in Table 1 (Tufaro et al., 2021).

**TABLE 1:** Patient and vessel characteristics ( n = 50, one vessel per patient).

Age, yrs		62.04 ± 11.74
Male		38 (76%)
Vessel location	LAD	35 (70%)
	LCx	7 (14%)
	RCA	8 (16%)
Vessel length		0.028 m ± 0.009 m
Proximal diameter		2.32 mm ± 0.4 mm
Degree of stenosis		47.0% ± 5.7%
Inflow velocity		0.13 m/s ± 0.02 m/s

#### 2.1.2 Computational fluid dynamics data

CFD simulations were conducted based on the 3D vessel geometries reconstructed from 3D QCA. The corresponding pressure and WSS were calculated over the lumen-wall interface of each patient, using the patient-specific inflow condition, itself estimated from the velocity of the contrast agent (derived from the length of the model, the time required for the contrast to fill the vessel, and the cine frame rate) and assuming steady state. Details of CFD modelling has been presented elsewhere (Tufaro et al., 2021). The anatomical and haemodynamic features of the vessels are summarised in Table 1.

The anatomical and CFD data were extracted over the lumen wall surface, resampled on a rectangular grid that has 36 data points circumferentially and segments at 1.5 mm increments longitudinally (i.e., 24–77 segments). This was conducted using a custom-made MATLAB code. The local anatomical feature map of each patient can be used as input in training and prediction of a model, whilst the CFD-derived variables (pressure and WSS) are the outputs. This paper focuses on the prediction of WSS only, because pressure drop prediction has not only been conducted more thoroughly using ML (Farajtabar et al., 2021; Fossan et al., 2021; Pajaziti et al., 2023), but can also be calculated reliably using reduced-order 0D models (Schrauwen et al., 2014).

We chose to use 7 input features from the original CFD data, as outlined in Table 2. Centreline-based features were mapped to the rectangular grid on the lumen border, i.e., points on the lumen border on one cross-section have the same centreline-based features. Going forward, we calculate additional morphological features and standardised the data format as described in the Feature Engineering section.

**TABLE 2:** Patient data features considered in DL model. Centreline-based features are mapped to corresponding locations on the lumen border.

Feature		Description
Input (from original CFD data)	$x_{w},y_{w},z_{w}$	XYZ coordinates of point on lumen border
	$x_{c},y_{c},z_{c}$	XYZ coordinates of centreline point
	v	inflow velocity
Input (added)	$r_{w},\theta_{w}$	Polar coordinates of points on lumen border
	c	Centreline curvature (Eq. 2)
	$d_{c}$	Distance along centreline
	$\vec{t_{cx}},\vec{t_{cy}},\vec{t_{cz}}$	Tangential vector of centreline
	$\vec{n_{cx}},\vec{n_{cy}},\vec{n_{cz}}$	Inward curvature vector of centreline
Output	$\tau_{\mathrm{wss}}$	WSS magnitude on lumen border, calculated from CFD

#### 2.1.3 Feature engineering

In addition to the features available as part of the original dataset, we calculated a variety of geometrical features that may improve the predictive power of the model. These features include the polar coordinates of the wall, centreline curvature, distance along the centreline, tangential and inner curvature vectors of the centreline. Each new feature is calculated as follows:

• Cylindrical coordinate: The polar coordinates include the radius $\left(r_{w}\right)$ and angle from reference starting point $\left(\theta_{w}\right)$ . All vessel models have their centreline, and $r_{w}$ was calculated by subtracting the $x_{c}y_{c}z_{c}$ coordinates of the centreline points from the $x_{w}y_{w}z_{w}$ coordinates of each point on the lumen border. Based on this, we obtain a reference unit vector $v_{\hat{r}ef}$ and a corresponding vector for each point $v_{\hat{t}mp}$ . $\theta_{w}$ was calculated using Eq. 1:

$$\theta_{w}=\frac{\arccos\left(v_{\hat{r}ef}\cdot v_{\hat{t}mp}\right)*360}{2\pi }$$ (1)

• Centreline Curvature: This is calculated using the Menger formula, as shown by Eq. 2. The Menger formula is applied to the centreline data for a patient, where $p_{i-1}$ , $p_{i}$ , and $p_{i+1}$ are 3 consecutive points along the centreline, R is the radius of curvature, and A reflects the area of the triangle that spans between $p_{i-1}$ , $p_{i}$ , and $p_{i+1}$ .

$$c\left(p_{i}\right)=\frac{1}{R}=\frac{4A}{\left|p_{i-1}-p_{i}\|p_{i}-p_{i+1}\|p_{i+1}-p_{i-1}\right|}$$ (2)

• Distance along centreline: This is calculated by taking the cumulative distance between each $x_{c}y_{c}z_{c}$ , centreline point and the inlet.

• Tangential and inner curvature vectors: the tangential vector of each point along the centreline was calculated by taking the coordinate difference of adjacent centreline points. The variation of normal vectors between adjacent centreline points was then calculated as the inner curvature. This adds 6 variables (3 components of 2 vectors).

The final set of input includes the original 7 features with 10 engineered features, 17 features in total.

#### 2.1.4 Data normalisation and imputation

As the cardiac anatomy varies between individuals, the various coronary arteries all of which have different contours and shapes were analysed together. As their Cartesian coordinates would not be expected to mapped onto each other, a common feature space had to be created. To make the data consistent, the proximal end of each vessel centreline was first shifted onto the origin (0, 0, 0). The vessels were then rotated to align the global centreline vector (i.e., the vector connecting the first and last point of the centreline) with the global *Z*-axis. This was applied onto its coordinates such that all geometries ultimately align in the same plane.

Furthermore, the number of slices for each patient ranged from 24 to 77. The training data needs to conform to a consistent shape for all patients. Accommodating the lowest common denominator would lead to a truncation and therefore loss of critical patient data. We therefore interpolate the patient data to the maximum number of slices (77 slices). Nonetheless, a U-net shaped architecture with pooling requires an even number for this dimension, thus we use 76 slices per patient. The increments at which the coronaries are sliced comprises in the range of 0.24–0.66 mm.

**FIGURE 1:**
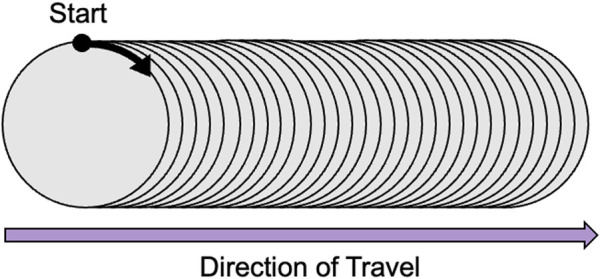
Data structure showing the data scanning direction: Starting point at θ=0 travels clockwise for each slice and along vessel from left to right.

The structure of the data that will be used for the DL model becomes an array in the shape ( n , 76, 36, 17) where n is the number of patients. Initially n is the set of the 50 patients; these will later be augmented with synthetic data. As for the remaining array dimensions: 76 is the number of slices, 36 is the number of points along the circumference of each slice (every 10 degrees), and 17 is the number of features for each point. As the output is a prediction of the WSS for each point, our output is in the following format: ( n , 76, 36, 1). Figure 1 depicts the structure of the data along with the direction of travel more clearly.

### 2.2 Synthetic data generation

Deep learning models typically need many samples for training. This is because a model will need to learn the weights for numerous parameters and uses gradient-based optimization to converge to a global optimum. When the model attempts to fit data patterns, it may learn random noise if a dataset is small. This causes what is known as overfitting and has as a consequence the inability of the model to generalise well. We have conducted a practical experiment to show that as the training data size increases, model loss decreases (refer to Supplementary Figure S9).The need for larger realistic datasets, and particularly those that are more private/secure (obfuscate real patient data), have driven studies into the generation of synthetic data. It is important that the synthetic data follows the underlying distribution of the real data and captures correlations between features in order to be plausible. As yet, there are no public datasets of arterial CFD that can be used for training. Since in our study we have only 50 patients in scope, it is essential to augment the training set by generating synthetic data.

In machine learning, and particularly in the case of images, there are many simple approaches that can be utilised to create a larger sample. These methods are referred to as data augmentation and involve transforming an image via shifting (horizontally or vertically), flipping (horizontally or vertically), rotating (clockwise or anti-clockwise), zooming in or out, and changing the brightness (Chlap et al., 2021). These methods can create more variation in the data and artificially expand the dataset.

The use of flipping as an approach of data augmentation is commonly seen in medical image processing (Cossio, 2023; Perez and Wang, 2017). Nishio et al. applied random 10° rotations, translations, and horizontal flipping to chest X-ray radiography (CXR) images in order to improve the accuracy of their CNN model for COVID-19 diagnosis (Nishio et al., 2020). Schmid et al. (2023) propose the use of statistical shape and intensity models (SSIM) to generate augmented CT images of hip bones including flipping, shifting and rotation. Although the reversal of inlet and outlet may not be physiologically representative, we examined the ratio of inlet and outlet radii, which is 0.86 ± 0.12 and suggesting the tapering is not substantial. Flipping remains a crucial step to avoid overfitting and creating a more general model.

Inspired by these approaches, we modified the geometry of the coronary artery and running the CFD analysis in ANSYS. In that way, we were able to create realistic synthetic data by making 3 modifications to the original patient data. After implementing the following modifications listed, 550 synthetic patients were created that were used for training.

• Reverse inlet and outlet boundaries: The inlet and outlet boundaries were reversed (i.e., flipping was applied) for the original patient data whilst maintaining the original patient inlet velocity. Thus, this created 1 new dataset per patient and overall 50 synthetic datasets.

• Modify inlet velocity: The velocity was modified for each patient to range from 0.05 to 0.25 m/s at 0.05 increments, aligning with ranges observed in literature (Marcus et al., 1999; Zafar et al., 2014). This resulted in 5 new datasets per patient and therefore 250 synthetic datasets.

• Combination: By combining the methods above and simultaneously changing both the inlet velocities while reversing the inlet and outlet boundaries, we were able to generate another 5 datasets per patient and therefore an additional 250 synthetic datasets.

### 2.3 Deep learning model and training

In order to train the model, we split the synthetic dataset generated. Of the total 600 patient dataset (50 real + 550 synthetic), 80% was used for training (40 real + 440 synthetic generated from those) and 20% was used as a test set (10 real patients). The validation set, still used as part of the training process for hyperparameter tuning, is automatically created once the training begins and comprises 20% of the training set (96 real/synthetic data mixture). It is important to note that the train/test split is completed at the real patient level, such that only synthetic data associated with the 40 real patients (440 patients) can be used for training. This is done in order to prevent leakage of information from the training set into the test set. In other words, the model has not seen any information similar to the test patients, not even their synthetic data. Once all data creation and pre-processing was complete, the neural network architecture was designed.

**FIGURE 2:**
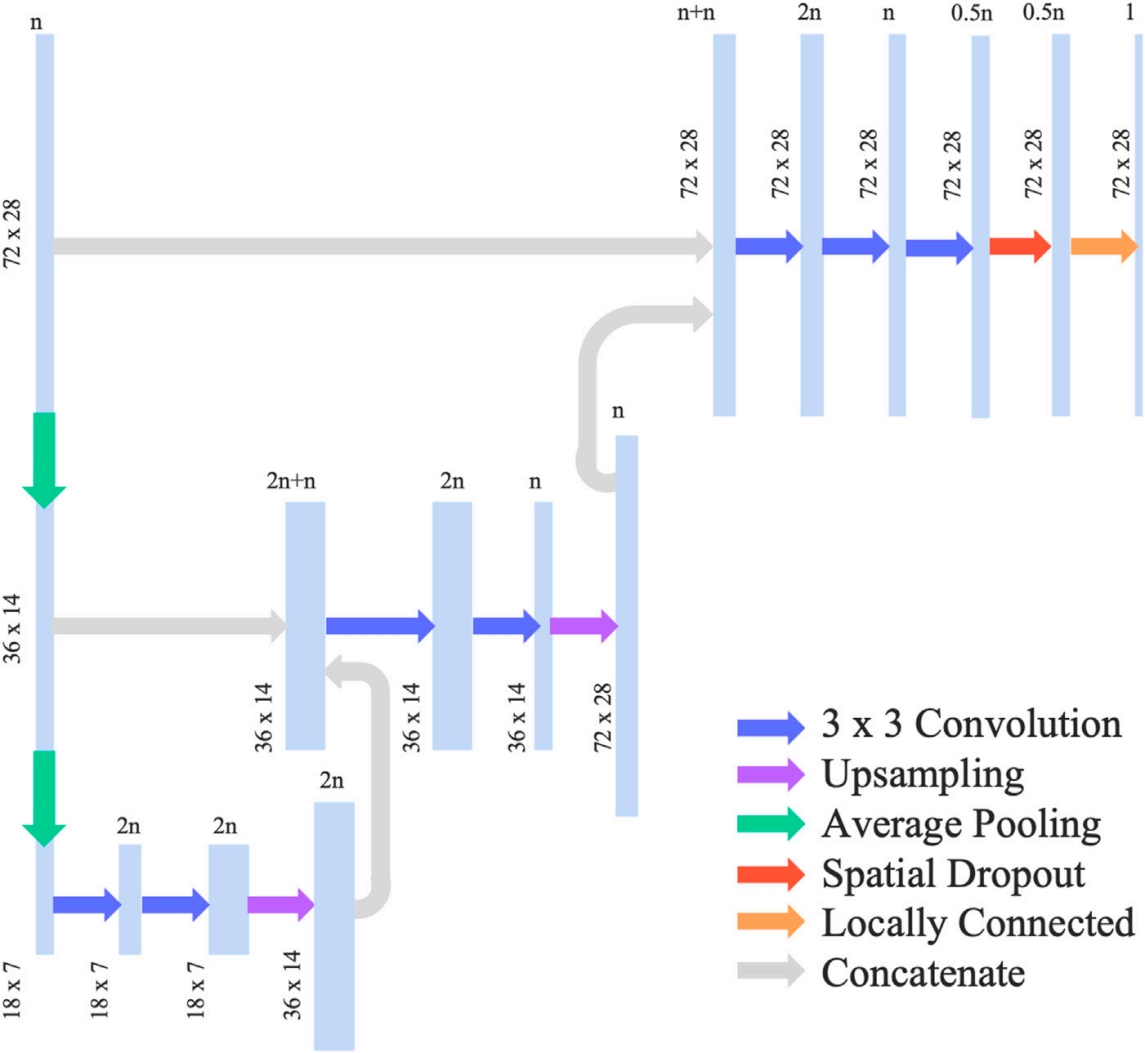
Neural network architecture for prediction of WSS in coronary artery.

We adopted and modified the neural network architecture used in Gharleghi et al. (2020) by removing the concatenation with global features such as bifurcation angle, which is not relevant for our use case. We also added a spatial dropout layer in order to reduce overfitting of the model. The final neural network can be categorised as a U-Net and had an architecture shown in Figure 2. This network uses average pooling to scale the input data down to half of its resolution. This is done twice, leading to a quarter representation of the original dataset. The data is then passed through 2 convolutional layers where a 3 × 3 convolution was applied, followed by up-sampling and concatenation with the higher resolution data. Adam optimizer was then used in addition to a 10% spatial dropout as a regularization technique. The activation function selected was ReLU such that the output is constrained to a positive value.

As the loss function, we selected the mean squared error (MSE) although other similar studies in the literature, including Gharleghi et al. (2020) and Jordanski et al. (2018), typically adopt the mean absolute error (MAE). The MSE was calculated as per Eq. 3, where n represents the number of vessels, $\tau_{i}$ represents the observed values (i.e., CFD-based WSS), and ${\hat{\tau }}_{i}$ represents the predicted values (i.e., DL-based WSS).

$$MSE=\frac{1}{n}\overset{n}{\underset{i=1}{\sum }}{\left(\tau_{i}-{\hat{\tau }}_{i}\right)}^{2}$$ (3)

In the case of our patient dataset, we are investigating patients with stenosis where WSS is significantly elevated. Our choice of MSE is to better predict the peak WSS, as it incurs a higher penalty when mismatched. To highlight the importance of this, we also trained the model using MAE for comparison. The model was trained for 1,000 epochs. The computational resource utilised was an NVIDIA T4 Tensore Core GPU with 52GB RAM. Training time is approximately 40 min on average. Increasing infrastructure resources can lead to faster model training and inference.

### 2.4 Model evaluation and investigation

The deep learning model was assessed for WSS prediction accuracy. We implement k-fold cross-validation with 5 folds, where the DL-based prediction of WSS for the 10 patient data left aside in Section 2.3 are compared against their original CFD-based WSS, over 5 repeated training ∼ validation steps.

DL models are typically considered as a black box models, and the associations between particular inputs and outputs have not been formally investigated. However, as our work also aims to understand the mechanistic insight behind WSS profiles, explainability (xAI) techniques were used to quantitatively assess feature importance on a trained model. Many techniques exist that have been used in DL models such as Shapley values (Lundberg et al., 2017), however as this approach is computationally expensive, and the model internals are available, we opted for the integrated gradients approach.

Integrated Gradients is a common technique for explaining differentiable models such as neural networks. It is based on two key properties: implementation invariance and sensitivity. It works by creating a straight path between a reference point (typically zeros) and the inputs to the model. By partitioning and interpolating the path, the model can compute predictions at the different partition points. The gradient information at the points of this path are calculated, making it computationally efficient (Holzinger et al., 2022). The intuition is that the gradient in the points where a model’s predictions have flattened out are zero and therefore do not contain information that contributes to the explanation. With this process, the significance of each input feature in the prediction of output (i.e., WSS) can be estimated. In order to apply this method on our deep learning model, we utilise the Innvestigate library^1^ (Alber et al., 2018).

## 3 Results

### 3.1 Overall model assessment

The results of the MSE on the test set are shown in Table 3. The mean absolute error (MAE) is also displayed to show how different the absolute value of the prediction is from the gold-standard, CFD-based WSS for the same patients. In the literature, the normalised mean absolute error (NMAE) is typically reported, thus we include this metric in order to provide comparison with the other state-of-the-art methods. The NMAE can be calculated by either dividing the MAE by the mean or by the difference between the maximum and minimum WSS. For this paper, we divided by max-min, where the difference in WSS is 33.33 Pa.

**TABLE 3:** Global model error evaluated for 10 test patients. The two groups of error values are based on different loss functions (MSE and MAE) used in training, and the error is also assessed based on both MSE and MAE. Summary statistics are provided for training patients for comparison ( ${Mean}_{tr}$ and ${SD}_{tr}$ ). Full error statistics of training can be found in Supplementary Table S4.

Fold	Models trained on MSE loss			Models trained on MAE loss		
	MSE ( ${Pa}^{2}$ )	MAE [Pa]	NMAE [%]	MSE ( ${Pa}^{2}$ )	MAE [Pa]	NMAE [%]
1	9.3	2.08	6.24	8.4	1.91	5.73
2	8.9	2.09	6.27	8.6	1.91	5.73
3	6.3	1.77	5.31	12.6	2.44	7.32
4	7.1	1.90	5.70	8.1	1.87	5.61
5	11.5	2.21	6.63	12.0	2.37	7.11
Mean	8.6	2.01	6.03	10.0	2.10	6.30
SD	2.01	0.18	0.47	2.17	0.28	0.75
Mean*_tr_*	7.3	0.88	2.65	11.7	1.23	3.68
SD*_tr_*	2.01	0.12	0.37	07.5	0.61	1.83

As previously mentioned, the model training with our data took on average 40 min. This means that it took less than 2.5 s per epoch; not impractically long in the current form, yet it can be accelerated even further in the future with additional infrastructure resources. Model training results are presented in the Supplementary Table S4. Prediction time was approximately 35 milliseconds, which is many orders of magnitude faster than the CFD processing time, which ranges from 20 min to approximately 3 h in our cases. Furthermore, the model was able to predict the WSS on the test set to a NMAE of 6.03% with a standard deviation of 0.47% when normalised by the difference between the maximum and the minimum WSS.

**FIGURE 3:**
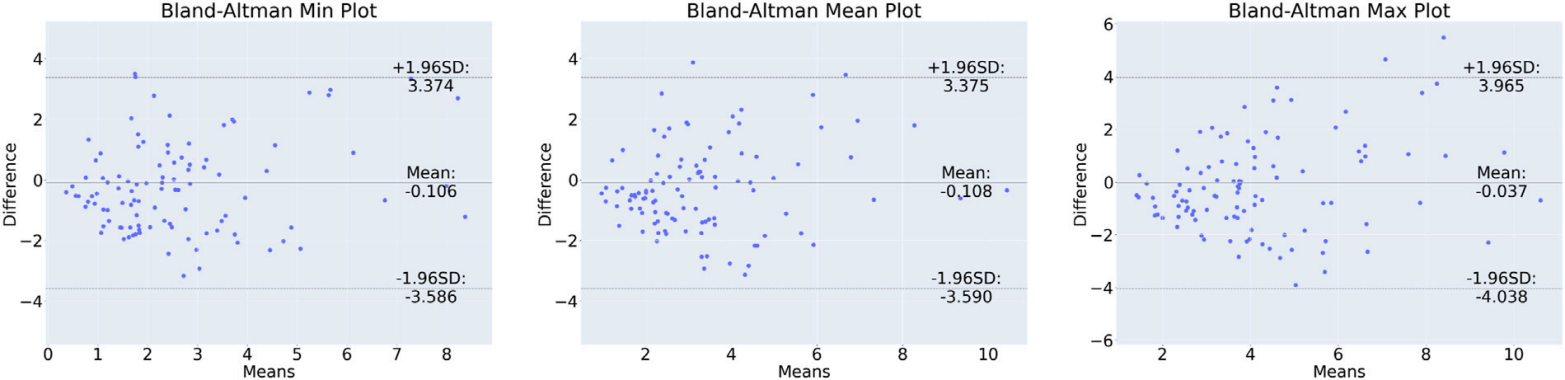
Bland-Altman plot for min (left), mean (centre) and max (right) predominant WSS in 3-mm segments along the vessels.

In order to show correlation between the CFD-based WSS and the DL model prediction, a Bland-Altman plot was generated for the test set and shown in Figure 3. Here, the minimum, maximum, and mean predominant WSS in every 3-mm vascular segments were used. Briefly, predominant WSS is defined as a moving average of WSS within a window around a point of interest, which has a size of 90° circumferentially and 3 mm longitudinally. These metrics were originally proposed to compensate image data uncertainty, associated with motion of an intravascular imaging catheter during a heartbeat, in the creation of a 3D vascular model. They have been used in previous studies examining the value of WSS in predicting adverse clinical events (Stone et al., 2018), including ours (Tufaro et al., 2021), and shown to be practical and effective. The means and differences are calculated between the CFD-based and DL-predicted WSS and plotted along with the ± 1.96 SD lines. Figure 3 shows the values for all the patients. The majority of points lie between 95% CI, demonstrating a correlation between the CFD-based and DL-based WSS values, with the bias of maximum 0.38 Pa.

### 3.2 Patient-level WSS prediction

**FIGURE 4:**
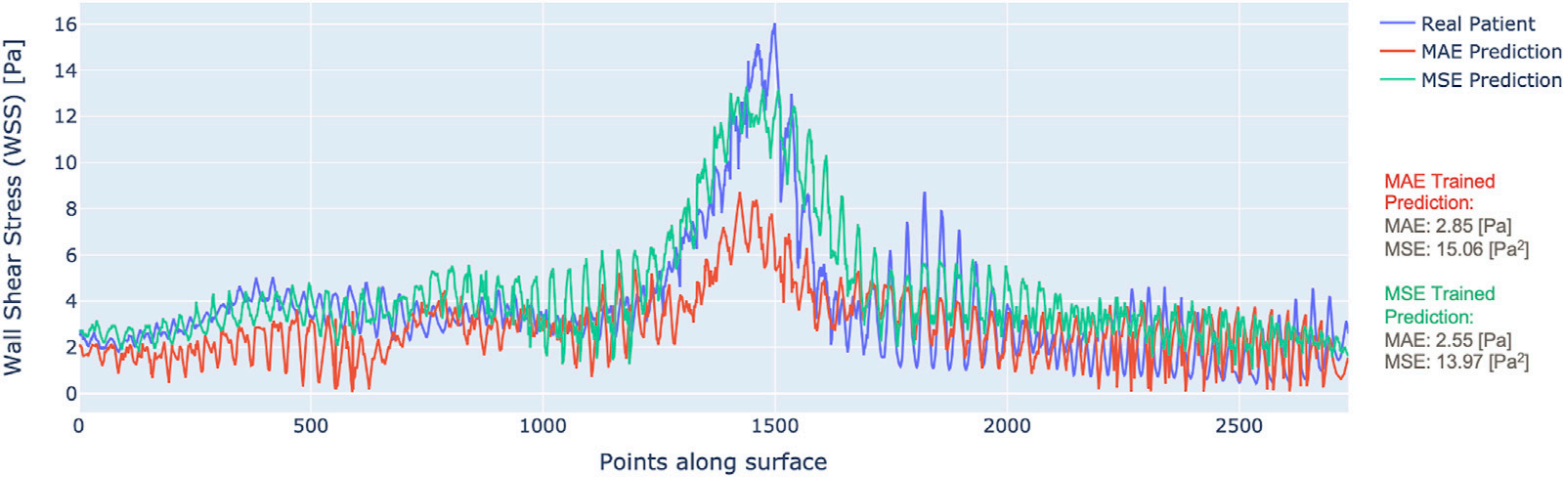
WSS profile for one patient with predictions from MAE (red) vs. MSE (green) trained models.

By scanning along the slices of data in the direction depicted by Figure 1, we can plot the WSS profile for a particular patient. Figure 4 illustrates the WSS profile for a patient in more detail, the DL-predicted WSS values are plotted over the wall surface from proximal (left) to distal (right) and across the circumference of each cross-section.

The plotted data display the output of the model trained with MAE as the loss (red) and the MSE as the loss (green), along with the ground truth CFD-based WSS that are displayed in blue. It is clear that the model trained by MSE predicts WSS that fits better to the ground truth. Although the MAE result had a low overall error, it did not capture the local variations of WSS as much as the MSE-based training, especially in the areas of stenosis. This justifies the choice of MSE loss for model training.

**FIGURE 5:**
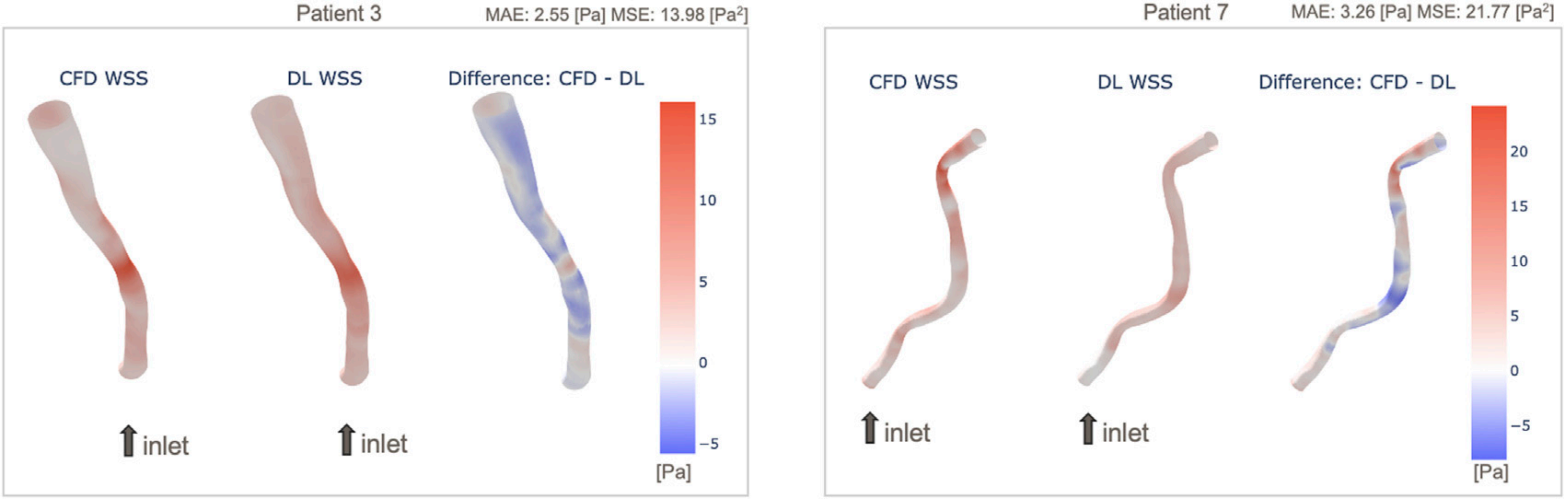
Example 3D maps of predicted WSS, in comparison with original CFD-based WSS: Patient 3 with small prediction error (left) and Patient 7 with larger error (right). WSS maps of all 10 patients are shown in Supplementary Figure S10.

Among the 10 test cases, we performed a qualitative assessment using 3D WSS surface plot in order to illustrate in more detail the patient-level WSS prediction results. Two of those vessels with the most accurate and inaccurate WSS predictions are presented in Figure 5, and similar plots for the full 10 test cases are presented in the Supplementary Figure S10. It is apparent that in both cases the WSS patterns are generally captured by the DL model, but there are differences after looking the data in detail, e.g., in the distal region of Patient 7 where the vessel is more tortuous. This trend is observed in the other models shown in the Supplementary Material. Patient 7 appears to be the most curved/tortuous and having multiple stenosis sites, which could have given the difficulty in the prediction of WSS by the DL model. Additionally, we observed that the prediction by the DL model underestimates WSS in the stenosis region and tends to overestimate otherwise.

### 3.3 Feature importance

**FIGURE 6:**
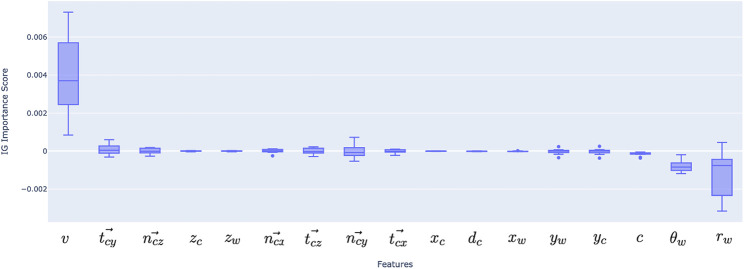
Feature importance for deep learning model inputs, aggregated over 10 test patients’ data. Positive and negative scores represent positive and negative correlations, similarly to statistical correlation coefficients.

Figure 6 shows the value of the 17 features based on the integrated gradients estimations. The integrated gradients are calculated per each data point on the vascular wall surface and give an evaluation on how the input features contribute to the prediction. In order to conduct an overall assessment of the model, the samples need to be aggregated; the mean feature importance across all points along a vessel surface, and the mean across all test patients are summed to have the final results. Our findings indicate that the radius and inlet velocity are the most important features while the circumferential coordinate θ , the components of centreline tangential and normal vectors $t_{x}$ and $n_{y}$ were found to be the less contributing features. The positive and negative signs indicate whether the relationship between the input feature and the output feature (WSS) is positively correlated or inversely proportional.

The top six important features were then selected (velocity, radius, theta, curvature, tangential vector in Y direction and normal vector in Z direction) and the model was retrained with MSE as the loss function. This resulted in an MSE of 8.89 ${Pa}^{2}$ , an MAE of 2.28 Pa, NMAE of 6.84%. As expected, the results are comparable to using the full set of features available. This is because the model is placing a low weight on the remaining features, as such they do not have a strong impact on the final prediction. Nonetheless, utilizing the full feature set (with MSE loss) results in an average MSE of 8.6 ${Pa}^{2}$ , an MAE of 2.01 Pa, and NMAE of 6.03%; thus still outperforming the experiment with the subset of features.

## 4 Discussion

In this study, we conducted DL-based WSS prediction of stenosed arteries based on CFD-based WSS calculations of 50 patients’ and 550 synthetic vessels. The results showed, that despite the relatively limited number of training data, DL-based WSS prediction is feasible in stenosed patient-specific geometries. WSS prediction of diseased coronary arteries is a challenging problem as the range of WSS that needs to be predicted is much larger than that of a vessel without a stenosis including bifurcations. Dolan et al. (2012) reported that the WSS of the arterial system ranges from 1 to 7 Pa while in straight arteries, the time-averaged WSS physiological range is between 1.5 and 2.5 Pa. In the areas of bifurcation, WSS is raised because of flow impingement on bifurcation carina and range from 11 to 34 Pa (Lindekleiv et al., 2010). However, in the areas of stenosis this can even be > 30 Pa (Teng et al., 2010; Torii et al., 2009), observed both in our CFD models (e.g., in Figure 4) and others.

### 4.1 Synthetic data

As mentioned earlier, training of DL models tend to require large data sets. For example, Cho et al. (2016) investigated the impact of increasing the number of training samples of axial Computed Tomography (CT) images for classification into six anatomical classes. The results showed that an increase in the number of samples from 5 to 200 improved model accuracy from 8% to 95% (Cho et al., 2016). Similar findings have been also reported in other studies that highlighted the relationship between a larger data set and improved model performance, not only for machine learning but especially for deep learning (Sarker, 2021; Halevy et al., 2009; Zhang et al., 2019).

Gharleghi et al. (2020) generate a synthetic dataset out of 127 patients by modifying the bifurcation angle geometry and, as a result, obtain 3,302 synthetic patients. In our paper, to augment our dataset, we took a simple approach by flipping the geometry in combination with changing the input velocity. This results in 550 synthetic patients that were then used to train a DL model that can predict WSS with an accuracy that is similar to previous reports. An advantage of the proposed method is that it does not require creation of vessel geometry and computational mesh generation. These are time-consuming processes requiring up to 3 h to estimate the WSS patterns of a single new synthetic “model.”

The training dataset size in this study, is deemed effective in terms of the trade-off between accuracy and training time, based on our preliminary investigation using idealised curved tubes with a stenosis (Supplementary Material). However, it is likely that a larger training datasets would provide more accurate estimation of WSS patterns as real coronary anatomy varies between patients and vessels and this critically determines WSS. To mitigate this, acquisition of real patient data is ideal, but considering the largest number of patients reported in a CFD analysis is approximately 500 (Stone et al., 2012), synthetic data will still be necessary. Additionally, patients’ data are often associated with confidentiality concerns, which may prevent the dataset from being shared with the research community. Thus, future studies should try to combine the inclusion of larger clinical datasets and the creation of synthetic data from these data.

A common method for synthetic data generation of geometries in biomedical engineering is statistical shape modelling (SSM). Three-dimensional (3D) SSM facilitates the study of shape variability and allow the creation of new geometries with a wide range of variability (Alemany et al., 2019). Such statistically varied models offer an opportunity for experiments by exploring how changes to the shape geometry impact other factors (Sarker, 2021), which has been utilised in combination with CFD (Lamecker and Zachow, 2016). For example, Bruse et al. (2017) show that SSMs can aid to improve cardiac device development by modelling hemodynamic and geometric boundary conditions in cardiac anatomy. These models have been shown to improve both the efficiency robustness and value of synthetic patient data (King et al., 2019).

### 4.2 Model results

As previously noted, the MSE was used as a loss function as it facilitates a better fitting of the model prediction to WSS peaks due to the larger penalty in these areas. This is ideal for our vessels as there are sharp increases of WSS in the areas of a tight stenosis, reaching up to 70 Pa, whereas normal WSS range is reported to be 1–7 Pa (Malek, 1999). The WSS prediction error from the model trained on MAE turned out to be 2.10 ± 0.28 Pa, with a NMAE of 2.51 ± 0.33% (Table 3). This value on average is comparable to that calculated when MSE is the loss (2.01 ± 0.18 Pa MAE, *p* = 0.60 vs. MAE of the models trained on MAE loss). Therefore, as far as global error metrics are used to evaluate model accuracy, a difference is not evident and that may be the reason why most of the previous works utilised the MAE for simplicity. Additionally, when models are trained on MAE, the standard deviation of error (MAE) tends to be larger than MSE-based training (0.28 Pa vs. 0.18 Pa) though that is not statistically significant (*p* = 0.19 for F-test of training outcome MAE variances in Table 3).

Bland-Altman plots were generated for the minimum, maximum and mean predominant WSS to assess the difference between the CFD-based and DL-predicted estimations (refer to Figure 2). The limits of agreement range from −3.59 to 3.37 Pa. These ranges may have implications when WSS is low, but in the case of prediction of WSS in stenotic vessels where WSS in our dataset reached over 70 Pa, this result shows that the DL performed well and may have a value for real time computation of the WSS. The heterogeneous distribution of the WSS data in the test set underscores the challenge of the prediction task. Of note at the lesion site, which is the most clinically relevant segment, the range of difference between DL and reference standard was −4.04–3.96 Pa and this is comparable to that reported by Tufaro et al. (2022) (−4.1–5.7 Pa) who compared the estimations of two CFD-based approaches (one conventional CFD analysis performed using the ANSYS software a CFD analysis performed by a dedicated software CAAS Workstation WWS) in models reconstructed by two different software (Medis vs. Pie Medical).

An example of patient-level WSS prediction in Figure 4 shows that the error values are comparable for both the MAE trained and MSE trained models (2.85 Pa vs. 2.55 Pa respectively) however the WSS profile of the MSE approached better the reference standard in areas of stenosis. Nonetheless, in order to identify the areas that may be more difficult for prediction, a difference 3D plot is generated in addition to 3D visualization for DL-predicted and CFD-based coronaries (refer to Figure 5). Although the DL prediction is able to capture the overall WSS distribution, it can be seen that in diseased segments, the prediction is typically lower than the CFD-based results and the error is higher. Nevertheless, our prediction accuracy is similar to other existing models that range from 1.6% to 10.1% NMAE (normalised by the maximum) (Su et al., 2020; Suk et al., 2022), despite the fact that we included distinct stenoses.

### 4.3 Feature importance

Explainability is one of the current key advancements in machine learning models, moving forward from the use of black-box models (Belle and Papantonis, 2020). It is important to explain why a model has made a particular prediction before applying this in clinical practice. In typical supervised learning, e.g., linear regression models, model coefficients associating the input features to prediction output can be used to explain the importance of a specific input feature. However, this is more challenging with DL approaches, as they are typically black-boxes. In this paper, we introduced engineered features based on domain knowledge in order to improve model prediction. Feature engineering plays a key role in model prediction (Heaton, 2016). Previous works have demonstrated a relationship between key features and WSS, such as curvature and velocity (van Oijen, 2005).

Our results indeed indicate that velocity carries significant importance, following the vessel radius. This makes a mechanistic sense since WSS on a tube wall is theoretically determined by the flow rate and radius assuming Newtonian fluid and parabolic velocity profile $\left(\tau =\frac{4\mu Q}{\pi R^{3}}\right)$ . Curvature as Menger curvature is not shown as significant, but the components of centreline tangential and curvature vectors $\vec{t_{x}}$ and $\vec{n_{Y}}$ were found to be relatively significant features, along with the circumferential position θ , indicating that the curved vascular geometry and relative location of the wall play a role in WSS distributions. Further research is needed towards this direction and better understand WSS distribution. For instance, different features may play a different role in disease-free and stenotic segments.

### 4.4 Limitations

This study was an initial attempt to predict WSS in patient-specific stenotic vessel geometries, hence it has limitations. First, the number of patient-specific vessels was limited to 50, of which only 40 were used for training. This also limited the range of stenosis degree and inflow velocity as presented in Table 1. Although the results indicated comparable predictive capability to the literature, this can be improved by taking advantage of the full set of patients (n=293) included in our previous study (Tufaro et al., 2021). A larger number of patients with clinical outcome data will facilitate further assessment of DL-based WSS prediction more towards its clinical utility. Furthermore, the current output of the model is the magnitude of the WSS; not a WSS vector which is of high interest in hemodynamics modeling in combination with their transient behaviour. Steady-state flow is also an assumption that was made; by extending the work to unsteady flow, time-resolved WSS behaviour can be learned. Addressing these points will allow the study to include multidirectional WSS metrics, such as Oscillatory Shear Index, transverse WSS and WSS topology, etc. (Morbiducci et al., 2020; Hoogendoorn et al., 2019; Kok et al., 2019).

Future work is also needed to improve model performance and optimise DL architecture. The U-net approach used in this study is a well established approach, but it has been developed in 2015 (Ronneberger et al., 2015) and since then several other DL approaches have been introduced in cardiovascular research such as physics-informed neural networks (PINNs) (Raissi et al., 2019) and graph-neural networks (GNNs) (Scarselli et al., 2009). These DL architectures may enhance model performance.

Improvements to data augmentation in terms of quality and quantity can also be introduced. For instance, additional features may be engineered such as torsion in order to enrich the data set and, as a result, the data quality. Moreover, a finer level of granularity such as more slices per patient may be beneficial for achieving more realistic results, in addition to generating a larger cohort of synthetic data. Finally, a further deep dive into explainability should be considered, in particular in the areas of stenosis compared to the rest of the artery.

## 5 Conclusion

This paper demonstrated for the first time that DL-based prediction of WSS is feasible and has overall high performance that is comparable with previously-reported studies based on idealised stenotic vessels. The model used for prediction is inspired by a U-net architecture and achieves state-of-the-art performance at 6.03% NMAE. Training time is under 2.5 s per epoch and inference is at the order of milliseconds, making this a fast solution and an attractive alterative to current CFD analysis. Furthermore, we demonstrate the impact of utilising the MSE rather than the MAE as a loss function for training. Finally, model performance is explained via ranked feature importance calculated using the integrated gradients method. Although the model currently provides inaccurate predictions for some patients and may not yet be applicable for clinical application, it appears that it has the potential to replace the CFD-based WSS computation in clinical practice, as it is computationally inexpensive and able to operate in real time.

## Data Availability

The datasets for this article are not publicly available due to concerns regarding participant/patient anonymity. Requests to access the datasets should be directed to the corresponding author.

## Footnotes

1
https://github.com/albermax/innvestigate
