# Supplementary material for: Rapid prediction of wall shear stress in stenosed coronary arteries based on deep learning
Source: Front Bioeng Biotechnol. 2024 Aug 12;12:1360330. doi: 10.3389/fbioe.2024.1360330 (PMC11345599; doi:10.3389/fbioe.2024.1360330)
Supplement: Supplementary file 1 [file Presentation1.pdf]

Rapid Prediction of Wall Shear Stress in Stenosed Coronary Arteries based on Deep Learning

Correspondence*:
Ryo Torii
r.torii@ucl.ac.uk

SUPPLEMENTAL MATERIALS

Patient Data Cohort

In this section, the vessel geometries for the 50 patients that are in scope for this paper are presented. This is displayed to illustrate the complexities of the geometries and the variations in features such as the radius, curvature, stenosis, torsion, etc. As the vessels are collected from multiple hospitals, they have different coordinates in space. They are therefore normalized and aligned onto the X-axis both for visualization in Figure 7.

WSS Prediction in Fully Synthetic Vessel Models

Separately from the DL-model training using the real patient vessel geometries in the main text, we also built a predictive DL-model for WSS in fully synthetic, uniformly curved vessel models with a different degree of stenosis. Example geometries are shown in Figure 8. Using these models for training, validation and testing following the same methodology described in the main text, we aimed to identify the minimum number of patients needed to train our model. Specifically, we investigated the impact of the number of vessel models in training on the mean squared error (MSE) prediction loss in a test dataset using a standard feed forward multilayer perceptron (MLP) and 4-fold cross-validation. Per each vessel model, geometry and inflow conditions were varied by assigning 6 parameters (inflow velocity, reference vessel diameter, degree, location and extent of stenosis, global vessel curvature) randomly chosen from a normal (Gaussian) distribution for physiological range, e.g. vessel reference diameter range of 2.0-4.0 mm.

The results, MSE versus the runtime (i.e. cost) required to train the model for the number of patients provided, are shown in Figure 9. By normalising the MSE and runtime values, we are able to plot the mean normalised values and find the intersection of the line of best fit of the 2 charts; calculated using the ordinary least squares method. Based on this, we determine that approximately 600 patients is the current trade-off between the loss and the training time.

The results are in line with expectations based on the literature. The model is more generalisable and less prone to over-fitting with a larger number of patients, therefore improving model prediction and reducing validation loss. However, the time to train the model increases with the number of patients, thus associating a cost to a higher accuracy. The results support our number of vessels for training, 480, as in a good range of loss-runtime trade-off, although it should also be noted that the real patients' vessel anatomy and WSS distributions are more complex, and are likely to require a larger number of training datasets.

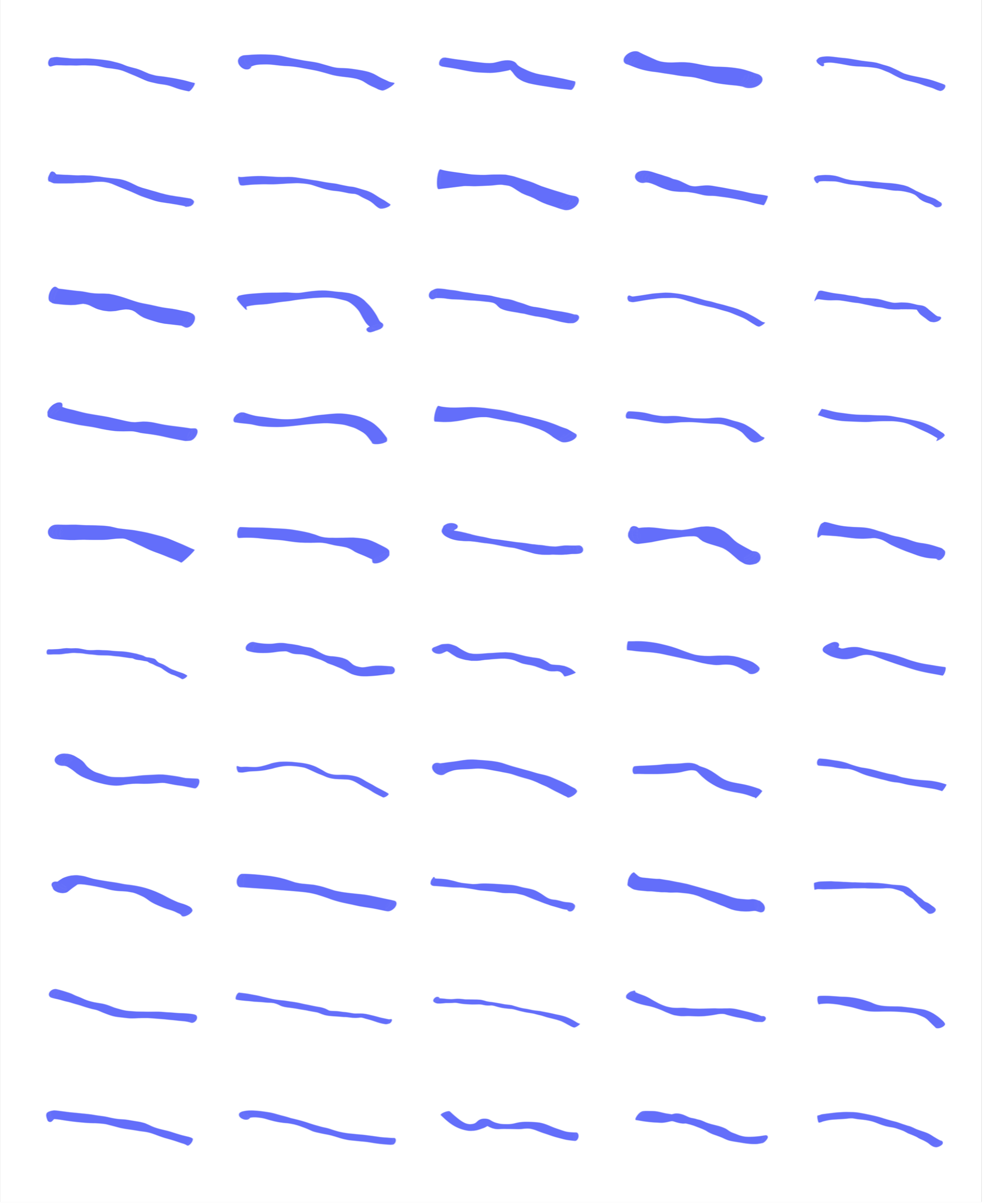

Figure 7. The patient data cohort comprised of 50 vessel geometries aligned to X-axis

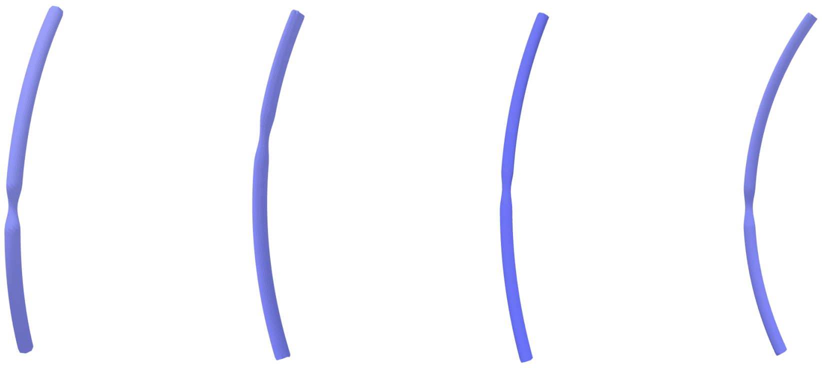

Figure 8. Example of 3D synthetic model tubes with stenosis. Patients vary in location, degree, and extent of stenosis, vessel diameter, global curvature, and inflow velocity.

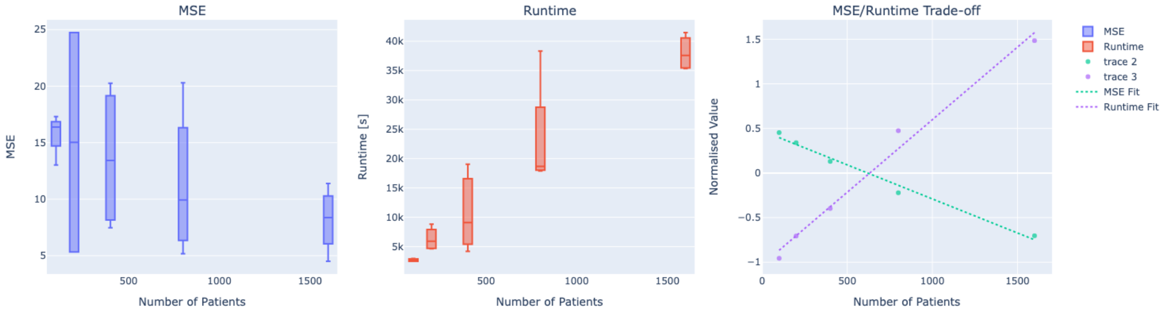

Figure 9. (MSE) MSE error for different number of patients. (Runtime) Time in seconds for training a model with different number of patients. (MSE/Runtime Trade-off) Mean MSE and mean runtime for each patient with line of best fit.

WSS Prediction in Patient Data Augmentation Training

In this section, the results of the error losses are displayed for the training set. These metrics can be compared with those presented in Table 4 in order to discern whether or not the model is overfitting. If the training error is much lower than the test error, then the model is not able to generalise well to an unseen dataset. Nonetheless, the errors are comparable between the two datasets, showing that the model is learning the best fit.

Table 4. Global model errors evaluated for training patients. The two groups of error values are based on different loss functions (MSE and MAE) used in training, and the error is also assessed based on both MSE and MAE.

	Models trained on MSE loss			Models trained on MAE loss		
**Fold**	**MSE**$_{tr}$ [Pa^2]	**MAE**$_{tr}$ [Pa]	**NMAE**$_{tr}$ [%]	**MSE**$_{tr}$ [Pa^2]	**MAE**$_{tr}$ [Pa]	**NMAE**$_{tr}$ [%]
1	4.9	0.95	2.85	8.0	0.99	2.97
2	5.1	0.75	2.25	8.9	0.97	2.91
3	9.9	1.07	3.21	26.5	2.44	7.32
4	7.9	0.90	2.70	5.5	0.81	2.43
5	8.9	0.75	2.25	9.6	0.92	2.76
**Mean**$_{tr}$	7.3	0.88	2.65	11.7	1.23	3.68
**SD**$_{tr}$	2.0	0.12	0.37	7.5	0.61	1.83

WSS Prediction in Test Patient Data

To provide an overview of model performance across a variety of geometries, the 3D maps of the test patient WSS CFD-based values are plotted alongside the predicted WSS values as per Figure 10 for all 10 test patients. These results are for Fold 4 of the model trained with MSE loss. This fold was selected as its error values are in-line with the average performance across the folds, making it the most representative.

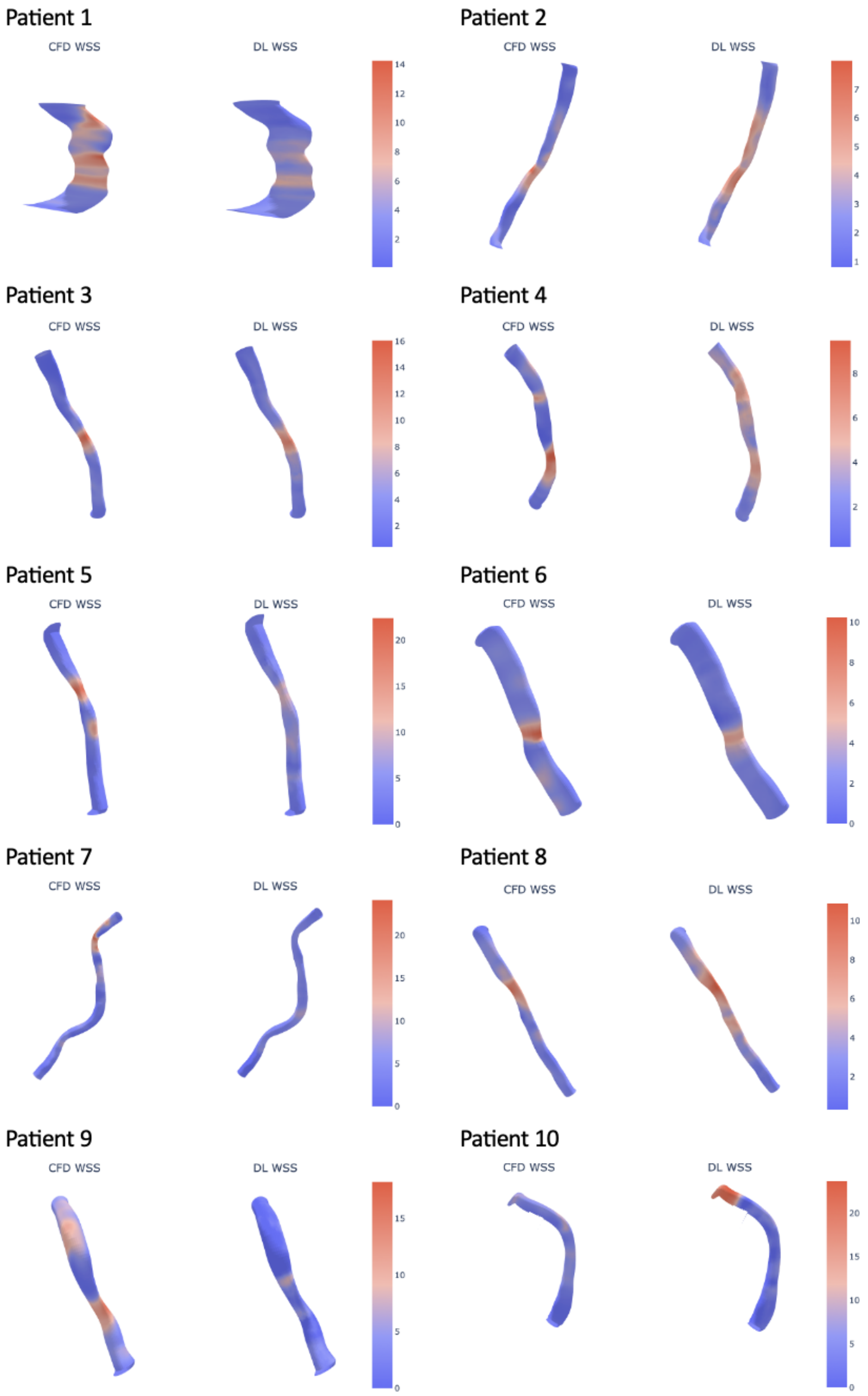

Figure 10. 3D maps of predicted WSS, in comparison with original CFD-based WSS for all 10 test patients. In each subfigure, the flow direction is always from the bottom to top of the figure.
